# Pollen exposure in pregnancy and infancy and risk of asthma hospitalisation - a register based cohort study

**DOI:** 10.1186/1710-1492-8-17

**Published:** 2012-11-07

**Authors:** Adrian J Lowe, David Olsson, Lennart Bråbäck, Bertil Forsberg

**Affiliations:** 1 Occupational & Environmental Medicine, Department of Public Health and Clinical Medicine, Umeå University, Umeå, Sweden; 2 Epidemiology and Global Health, Department of Public Health and Clinical Medicine, Umeå University, Umeå, Sweden; 3 Murdoch Children’s Research Institute, Melbourne, Australia; 4 Centre for Molecular, Environmental, Genetic and Analytic Epidemiology, School of Population Health, The University of Melbourne, Melbourne, Australia; 5 Department of Research and Development, Västernorrland County Council, Sundsvall, Sweden

**Keywords:** Pollen, Early life exposure, Asthma, Hospitalisation

## Abstract

**Background:**

A seasonal effect of month of birth and risk of allergic disease has been suggested by numerous studies. Few studies have directly measured pollen exposures at different points during pregnancy and in early life, and assessed their effects on risk of respiratory disease outcomes.

**Methods:**

Pollen exposure was calculated for the first and last 12 weeks of pregnancy and the first 12 weeks of infancy for all children conceived by women residing in Stockholm, Sweden, between 1988 and 1995. Hospital admission data for respiratory conditions in the first year of life was also collected.

**Results:**

Out of 110,381 children, 940 had been hospitalised for asthma by 12-months of age. Pollen levels showed both marked seasonal variations and between year differences. Exposure to high levels of pollen in the last 12 weeks of pregnancy was associated with an increased risk of asthma hospitalisation (aOR = 1.35, 95% CI = 1.07-1.71 for highest quartile versus remaining infants). Exposure to high levels of pollen in the first three months of life was associated with a reduced risk (aOR = 0.76, 95% CI = 0.59-0.98) but only in children of heavy smoking mothers.

**Conclusions:**

High levels of pollen exposure during late pregnancy were somewhat unexpectedly associated with an elevated risk of hospitalisation for asthma within the first year of life.

## Background

The role of allergen exposure in pregnancy and early life as a risk factor for subsequent allergic disease has been the subject of much debate. While observational studies suggest that high levels of exposure to house dust mite (HDM) allergen increase risk of allergic disease [1,2], intervention studies that reduce HDM allergen levels have failed to show any reduction in asthma or allergic disease outcomes [3-5]. Similarly, there is insufficient evidence to either recommend pet keeping, or removal, for prevention of allergic disease [6,7]. It is currently unclear if allergen exposure during pregnancy and early life may help induce tolerance, or promote allergic sensitisation and disease.

A number of studies have identified that pregnancy or birth during a pollen season is associated with increased risk of allergic sensitisation and disease [8-14]. In these studies season of birth has been used as a marker of pollen exposure, rather than actual measurement of concentrations of pollen [9,10,15]. Although the pollen season is a regular event each year, the timing, duration and intensity are dependent mainly on a range of meteorological, but also species and habitat factors, and there is substantial variability between years [16]. Although the associations seen in these studies have been largely attributed to pollen exposures, there are a range of other factors, including respiratory tract infections, residential moisture and pollution levels which also have strong seasonal variations, that could result in the observed associations. Very few studies have looked at the actual intensity of pollen exposures during specific time windows within early life as predictors of wheeze [17], asthma and other allergic diseases.

The aim of this register based cohort study was to assess the relationship between level of exposure to pollen during pregnancy and infancy and the risk of the child requiring hospitalisation for asthma.

## Methods

This study was based on Swedish public registers held by the National Board of Health and Welfare (Swedish Medical Birth Registry and Inpatient Registry)*,* the Swedish Museum of Natural History (daily pollen measurement) and the City of Stockholm Environment and Health Administration (pollution measurement). Information from the birth and inpatient registries were linked using the Swedish identification number (unique ten digit number assigned at birth or immigration), while daily pollen and pollution levels during pregnancy and infancy were estimated based on each child’s date of conception and date of birth. The study was approved by the Regional Ethical Review Board in Umeå.

### Study population

All vaginally delivered singleton births in the Greater Stockholm area from 1/1/1989 to 1/10/1996 were included in the study (n = 110,381). There were approximately 1.1-1.2 million inhabitants in this area over the study period. Information was extracted from the Swedish Medical Birth Registry, including date of birth, parity, birth-weight and length, gestational age, infant gender, and smoking habits of the mother during pregnancy. Gestational-age was calculated based on date of birth and estimated date of conception, according to ultrasound measures in early pregnancy (week 10–18) in 70.1% and maternal report of last menstrual period for the remaining pregnancies.

### Pollen and pollution exposures

The primary exposure was pollen concentrations in early life. Pollen levels were obtained from the Swedish Museum of Natural History. Daily concentration of total ambient pollen (pollen from all species combined), measured as pollen per m^3^ of air, for the region was measured using a single Burkard trap, located on the roof of the Palynological Laboratory (20 meters above ground) at Stockholm University, central Stockholm using methods that have been previously described [18]. For each child, the mean pollen level (sum of period specific daily pollen counts/84 days) for three periods: the first 12 weeks and the last 12 weeks of pregnancy, and the first 12 weeks of infancy, were calculated based on both the date of conception and date of birth. The City of Stockholm Environment and Health Administration provided data on the daily levels of NO_2_, O_3_, temperature and relative humidity, and period means were calculated.

### Hospital presentations for asthma and lower respiratory tract infection

All hospital admissions for asthma (ICD-9 coded 493) were collected from the Swedish Inpatient Registry for the period 1/1/1989 until 31/12/1997. Hospital admission for asthma within the first year of life was the primary outcome for this study. Details on the frequency of admissions during this time for lower respiratory tract illness (LRTI - ICD-9 codes 490, 491C and 491X) were also obtained from the Inpatient Registry, which was used as a marker for degree of potential exposure to respiratory pathogens in the first three and six months of life (sum of admissions for these diagnosis).

### Statistical methods

Logistic regression models were used to assess the associations between average pollen exposure for each time period and risk of hospitalisation for asthma. Linear associations are expressed as the effect per inter-quartile range increase in average daily pollen exposure for each exposure period (first = 93, and last trimester = 124, and infancy = 146 grains/day/m^3^ of air). Potential non-linear effects were assessed using the “fracpoly” (fractional polynomial regression) command within Stata. Effect modification was assessed using the likelihood ratio test for infant gender and maternal smoking history during pregnancy. Potential confounding effects of the following factors was assessed; season and year of birth and air pollution concentrations, and temperature and humidity. In addition, the model was adjusted for infant gender and maternal smoking during pregnancy. To assess if the effect of the pollen exposure varied between years, for each year of the study, the effect of high levels of pollen exposure (top 25% versus remaining children for that year for each time period) on hospitalisation for asthma in the first year of life were calculated separately, after adjusting for infant gender, gestational age, maternal smoking and season of birth. Results across the years were pooled using a fixed effects model using the inverse variance method [19]. The I^2^-statistics was used to assess the heterogeneity of the associations across the years [20]. All analysis was performed using Stata (release 10.1, Stata Corporation, College Station, Texas, USA, 2005).

## Results

**Figure 1:**
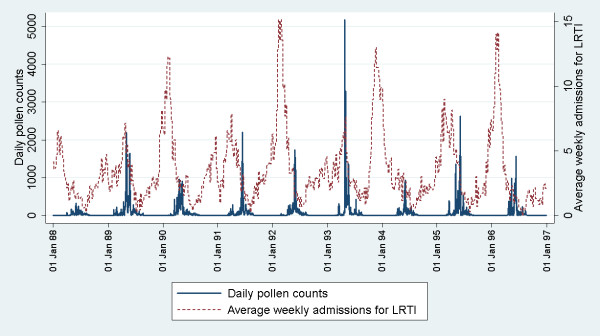
**Pollen counts (per m**^**3**^**of air) and weekly admission rates for lower respiratory tract infections during the study period.**

The daily pollen exposure varied dramatically, with a very high peak occurring in 1993 (Figure 1), and relatively low peak pollen days during 1988 and 1994. The peak periods of pollen also shifted from year to year (earliest 21^st^ April for 1990, and latest was 14^th^ June for 1991). There were also large variations in the rate of admissions for LRTI during the study period, but there was generally a low rate of admissions during the summer months (June-August) and peaking in January to February (Figure 1). The majority of the children were born to non-smoking mothers, and 0.85% of children were admitted to hospital for asthma by 12-months (Table 1).

**Table 1:** Demographic and pregnancy related details for the study population (n = 110,381)

		
Maternal smoking during pregnancy*	None smoker	75.7%
	1–9 cigarettes/day	10.5%
	≥10 cigarettes/day	6.4%
**Parity**	1^st^ born	47.7%
	2^nd^ born	34.6%
	3^rd^ born	12.6%
	4^th^ or subsequent	5.1%
**Birth details**		
Proportion of females		48.8%
Median (IQR**) gestational age (in weeks) at delivery		40 (39–41)
Median (IQR**) birth weight in kg		3.5 (3.2-3.8)
**Child outcomes**		
Proportion of children ever hospitalised for asthma		0.85%

* Details on smoking history missing for 7.3% of mothers.

** IQR – inter-quartile range.

Children who were born from February to July had the lowest risk of hospital admission for asthma (Table 2), while children who were born in October-November had the greatest risk (Table 2). Interestingly, this season of birth effect was present for some years but not others (data not shown). Adjustment for standard potential confounders did not greatly alter these associations, while there was some evidence that exposure to high periods of LRTI in infancy may explain the elevated risk of admission for asthma associated with birth in October-November. The drop in the association between season of birth and risk of asthma admission was greater when adjusted for pollen exposures.

**Table 2:** Associations between month of birth and risk of hospitalisation for asthma by 12-months

	**Crude**	**Standard adjustment**		**Adjustment for LRTI exposure**		**Adjustment for pollen exposure**		**Adjustment for both LRTI & pollen exposure**	
**Season of birth**	**OR**	aOR*	**Δ†**	aOR**	**Δ†**	aOR**	**Δ†**		**Δ†**
**Feb/Mar**	1	1	-	1	-	1	-	1	-
**Apr/May**	1.01 (0.81-1.27)	1.02 (0.81-1.28)	0.7%	1.00 (0.78-1.28)	−1.4%	1.09 (0.84-1.41)	7.8%	1.08 (0.83-1.41)	6.6%
**Jun/Jul**	1.05 (0.84-1.32)	1.06 (0.85-1.34)	1.1%	1.03 (0.80-1.31)	−2.6%	0.78 (0.57-1.05)	−26.3%	0.78 (0.58-1.06)	−25.5%
**Aug/Sep**	1.11 (0.89-1.40)	1.15 (0.91-1.44)	3.3%	1.04 (0.78-1.39)	−6.3%	0.86 (0.63-1.19)	−22.3%	0.88 (0.62-1.25)	−20.9%
**Oct/Nov**	**1.63 (1.31-2.02)**	**1.59 (1.28-1.97)**	−2.5%	**1.41 (1.03-1.93)**	−13.5%	1.33 (0.95-1.87)	−18.0%	1.35 (0.91-2.00)	−17.2%
**Dec/Jan**	**1.30 (1.04-1.62)**	**1.25 (1.00-1.56)**	−3.6%	1.16 (0.87-1.55)	−10.6%	1.16 (0.81-1.66)	−10.5%	1.15 (0.79-1.70)	−10.9%

All ORs are in comparison to children born in February to March (the period with the lowest observed risk).

* Adjusted for year of birth, maternal smoking, infant gender and gestational age.

** Also adjusted for pollen exposure (as a linear exposure) during the first and third trimester and during the first three months of infancy.

**†** All changes are relative to the unadjusted ORs in the table above. Positive values indicate an increase in the association, while negative values indicate a reduction in the OR.

In the crude analysis high pollen exposure in the last trimester of pregnancy was not associated with risk of hospitalisation (Table 3). However, when this association was adjusted for season of birth, high pollen exposure in late pregnancy was related to an increased risk of asthma hospitalisation. As such, season of birth appears to negatively confound (masks) the association between high pollen concentration in late pregnancy and increased risk of hospital admission for asthma in early life. The increased risk associated with high pollen exposure in late pregnancy strengthened when adjusted for measures of pollution, and was not altered by adjustment for rate of LRTI hospitalisations. Although the effect of high exposure to pollen in late pregnancy was consistent between years (I^2^ = 0%), 1993 & 1996 were the only years to demonstrate a clear increased risk. There was no evidence of non-linearity in these associations, nor was there evidence of interactions with pollen exposures and gender, or parity (p for all interactions >0.15).

**Table 3:** Associations between pollen exposures and risk of hospital admission for asthma by 12-months

	**Crude OR**	**Mutually adjusted**	Model 1*	**Model 2†**	**Model 3‡**
Effect per inter-quartile** range increase in pollen exposure					
**First trimester**	1.03 (0.98-1.09)	1.01 (0.95-1.07)	0.96 (0.88-1.05)	0.98 (0.89-1.08)	0.96 (0.88-1.05)
**Last trimester**	1.01 (0.93-1.09)	0.98 (0.90-1.08)	1.12 (0.97-1.29)	**1.18 (1.00-1.38)**	1.14 (0.98-1.33)
**Infancy**	**0.82 (0.74-0.90)**	**0.82 (0.74-0.90**)	**0.84 (0.70-0.99)**	**0.80 (0.65-0.97)**	0.84 (0.70-1.01)
**Highest 25% of children exposed versus remain children**					
**First trimester**	1.05 (0.91-1.22)	0.97 (0.82-1.14)	0.88 (0.70-1.11)	0.87 (0.67-1.12)	0.86 (0.68-1.09)
**Last trimester**	1.07 (0.92-1.24)	0.96 (0.81-1.14)	**1.34 (1.06-1.69)**	**1.44 (1.11-1.86)**	**1.35 (1.07-1.71)**
**Infancy**	**0.72 (0.61-0.84)**	**0.70 (0.59-0.84)**	**0.75 (0.58-0.97)**	0.77 (0.58-1.01)	**0.76 (0.59-0.98)**

* Model 1. Adjusted for year and season of birth, maternal smoking, infant gender and gestational age.

** Effect per inter-quartile range increase specific for period (first = 93, and last trimester = 124, and infancy = 146 grains/day/ m^3^ of air).

**†** Model 2. As per model 1, and also adjusted for averaged concentration levels of airborne pollutants (O^3^ & NO_2_) during the first and third trimester and during the first three months of infancy.

**‡** Model 3. As per model 1, and also adjusted for rates of hospitalisations for lower respiratory tract infections in first three months, and from three to six months of life.

In contrast, the high potential exposure to LRTI (as measured by rate of hospital admissions) during infancy was related to increased risk of hospitalisation for asthma in the crude analysis, but adjustment for season and year of birth reduced these associations (Table 4). Further adjustments for pollution and pollen levels did not alter these associations. Again, there was no evidence of non-linearity in these associations, nor was there evidence of interactions.

**Table 4:** Associations between circulating exposures to wheeze inducing pathogens, and risk of hospital admission for asthma by 12-months

	**Crude OR**	**Mutually adjusted**	Model 1*	**Model 2†**	**Model 3‡**
Effect per inter-quartile range increase**					
**First 3- months**	**1.15 (1.07-1.23)**	**1.16 (1.08-1.25)**	1.04 (0.93-1.17)	1.05 (0.92-1.20)	1.04 (0.91-1.17)
**3-6 months**	**1.17 (1.09-1.26)**	**1.19 (1.10-1.27)**	1.07 (0.95-1.20)	1.06 (0.93-1.20)	0.98 (0.86-1.12)
**Highest 25% of children exposed**					
**First 3- months**	**1.19 (1.04-1.38)**	**1.23 (1.06-1.42)**	1.06 (0.88-1.27)	1.07 (0.88-1.30)	1.07 (0.89-1.29)
**3-6 months**	**1.16 (1.00-1.34)**	**1.19 (1.03-1.38)**	0.99 (0.81-1.20)	0.96 (0.78-1.17)	0.98 (0.80-1.18)

* Model 1. Adjusted for year and season of birth, maternal smoking, infant gender and gestational age.

** Per inter-quartile range increase in hospital admissions for LRTI (18.7 admissions per week for both time periods).

**†**Model 2. As per model 1, and also adjusted for averaged concentration levels of airborne pollutants (O^3^ & NO_2_) during the first and third trimester and during the first three months of infancy.

**‡**Model 3. As per model 1, and also adjusted for period prevelences of pollen exposure in the first and third trimester, and the first three months of infancy.

High levels of pollen exposure in infancy were associated with reduced risk of hospitalisation for asthma in the first year of life (Table 3). Exclusion of 312 children who were admitted prior to three months did not greatly alter this association (aOR = 0.71, 0.52-0.98 for model 1). The protective effect of high pollen exposure in infancy was only apparent in children of mothers who were heavy smokers (aOR = 0.52, 95% CI = 0.33-0.82 for women smoking ≥10 cigarettes day). In contrast, there was no association between high pollen exposure in infancy and risk of hospitalisation for asthma in children of non-smoking mothers (aOR = 0.96, 95% CI = 0.75-1.24, p for interaction = 0.01).

## Discussion

In this exploratory analysis of a large, population based, cohort of infants born in Stockholm, Sweden, we observed that children whose mothers were exposed to high levels of pollen during late pregnancy had an increased risk of hospitalisation for asthma, while children exposed to high levels of pollen in infancy had a reduced risk, but only in children of mothers who were heavy smokers. The potential mechanisms to explain the protective effect of high pollen exposure in children of mothers who smoked are unclear. We speculate birth during high pollen periods would increase the likelihood of pleasant weather, allowing the mother and newborn to spend substantial periods of time outdoors, and thus reducing the degree of passive smoke exposure in these children. Future research is needed to confirm this effect.

The primary question with regards to these results is if exposure to high levels of pollen in late pregnancy might be causal for increased risk of early life asthma, or is this association due to residual confounding? We have attempted to adjust for a range of potential confounders for which data were available (specifically maternal smoking, sex, gestational age, and season of birth). Interestingly, this effect was only apparent when adjusted for season of birth. That is, the seasonal effects masked this increased risk. As exposure to pollen at various stages of pregnancy and infancy is almost a random event, many factors (including but not limited to pet keeping, infant diet and day care attendance) cannot plausibly be considered to be potential confounders of this association. However, there remains the possibility that other factors that show a correlation with pollen levels may have created these associations. Both low vitamin D levels in the child and the mother [21], and early life respiratory tract infections [22-24] have a strong seasonal variation, and have been associated with elevated risk of wheeze and asthma in the child. If these events occur at a consistent time following the pollen season, when children are most vulnerable to these exposures, then a spurious association with pollen levels could be created. However, it should be noted that 1) we have adjusted for season of birth and markers of pollution 2) the pattern of results is based on actual pollen exposure rather than season, and 3) the associations appear to be consistent across the years of this study. Furthermore, we have used hospitalisation data for LRTI as a crude proxy for the rate of circulating respiratory pathogens that may cause wheezing illness in the child, and this did not alter the associations with pollen.

If the association between high maternal pollen exposure and increased risk of hospitalisation for respiratory illness is causal, then these results are consistent with a potential priming of either the neonatal immune, or respiratory system, towards a wheeze/asthma like phenotype. Season of birth is associated with a range of differences in cord blood cytokine production profiles [25,26], and pollen may influence these patterns. It is likely that pollen sensitised mothers exposed to high levels of pollen during pregnancy are at increased risk of symptoms and asthma exacerbations. This may in turn change the intrauterine environment, predisposing the child to a Th2 type response. Alternatively, symptomatic mothers during high pollen periods may have increased risk pregnancy complications, including pre-eclampsia, preterm birth and impaired foetal growth [27], which could influence the risk of wheezing illness [28,29]. Unfortunately, we do not have information concerning maternal asthma or pollen allergy in this data set to test this possibility. Further work is required to elucidate exactly how pollen exposure may prime the foetal immune system towards severe respiratory illness in early life.

### Comparison with prior literature

The observed associations within this study may appear to be in conflict with prior literature on this topic. Kihlstrom et al. used data from 189 children born during the very high, 1993 pollen season in Sweden (data also included in this study) to argue that post-natal (0–3 months) exposure to pollen is more important than pregnancy exposure for the outcome of sensitisation to birch, and that maternal pollen allergy is more important than pregnancy pollen exposure for the outcome of childhood allergic rhinitis [11,30]. These results could be seen as demonstrating that pregnancy exposure is less important than infancy exposure. However, the work of Kihlstrom also indicates that a high pregnancy exposure is associated with a small increase in risk of asthma (OR = 1.3, 95% CI-0.8-2.1), when compared with both high neonatal exposure, and low exposure to pollen [11]. The pregnancy exposure group of Kihlstrom et al. included children who had high pollen exposure from approximately 13–31 weeks gestation [11,30], combining exposures during 2^nd^ and 3^rd^ trimesters. Hurley et al. observed an association with high pollen exposures during infancy and increased risk of wheeze up to two years of age [17], but did not examine the effect of pollen exposure during pregnancy. It is important to note that these previous studies have examined the outcome of asthma and wheeze, rather than hospitalisation for asthma, which may explain the differences between study findings.

This study has a number of important strengths and limitations that should be considered when interpreting these results. The study’s key strength relates to its large sample size of mother/baby pairs that have been assessed over multiple years. This provides both statistical power, and the ability to assess these associations over multiple pollen seasons. Furthermore, rather than relying on season of birth we were able to assess the associations with actual ambient pollen counts. The pollen count data allowed us to take into account variations in the initiation, duration and intensity of each pollen season between years. The outcome measure of hospitalisation for asthma (ICD-9 code 493) in early life is important for the health care system and the quality of life of affected children and families. Wheezing symptoms are common in infants, but it is very difficult to determine the nature of wheezing illness in young children, and many of the admitted children will not have symptoms that persist into later childhood. As such, it is likely that children in this study who have been hospitalised for “asthma” do not truly have this condition, but another form of respiratory condition. In Sweden, a diagnosis of “asthma” has been recommended for children aged less than 2 years with persistent wheezing symptoms or after at least three wheezing episodes on separate occasions in early childhood [31]. Also, it is more likely that diagnosis of asthma will be given if the child also has eczema or heredity for asthma (parents or siblings with asthma). Conversely, many children with wheeze in early life (that may be early onset asthma) are not hospitalised, but may be seen in outpatient setting. Unfortunately, we did not have access to data on outpatient visits for wheeze or asthma or use of inhalant therapy.

Also, we did not have access to information relating to other allergic disease phenotypes, including specific IgE and allergic rhinitis, pollen allergy and asthma at an age when the diagnosis is more reliable, all of which may have proven to be more informative. The measurement of pollen exposure (total pollen counts per day) was coarse, being captured in only one location, and it did not capture the species of the pollen or details of the smaller allergen carrying molecules [32], which may have resulted in misclassification of pollen exposure. The maximum distance from the pollen trap for those children included in this study was 17 km. The peak concentrations at this location are typically driven by high levels of birch pollen. Misclassification of pollen exposure will have biased associations towards the null, making it more difficult to observe any true associations. Future studies should attempt to make more personalised pollen exposure assessments. We also did not have data relating to early life respiratory tract infections for the actual child, nor to maternal asthma and allergic sensitisation, both of which have been proposed to interact with pollen exposure to influence risk of allergic disease [9,30]. We are currently planning a study to address some of these limitations.

## Conclusions

Replication of these findings is needed, as the results are somewhat surprising. Ideally future studies will need to have a sufficiently large sample size of children, who are born over a period spanning multiple years to allow for variability in pollen season and viral exposures, and to examine the effect of pollen exposure on a range of allergic disease outcomes into later childhood. If replicated, these findings may lead to mechanistic studies that help elucidate the pathogenesis of late pregnancy pollen exposure on respiratory outcomes, which could have therapeutic implications.

## Competing interests

All authors declare that they have no competing interests.

## Authors’ contributions

All authors participated in the design of the study. AL performed the statistical analysis and wrote the first draft manuscript. BF conceived of the study, obtained funding for collection of pollen data and participated in its design and coordination and helped to draft the manuscript. All authors read and approved the final manuscript.
